# Postural-respiratory function of the diaphragm assessed by M-mode ultrasonography

**DOI:** 10.1371/journal.pone.0275389

**Published:** 2022-10-10

**Authors:** Martin Sembera, Andrew Busch, Alena Kobesova, Barbora Hanychova, Jan Sulc, Pavel Kolar

**Affiliations:** 1 Department of Rehabilitation and Sports Medicine, Second Medical Faculty, Charles University and University Hospital Motol, Prague, Czech Republic; 2 Department of Health and Human Kinetics, Ohio Wesleyan University, Delaware, OH, United States of America; Ningbo University, CHINA

## Abstract

**Objectives:**

The diaphragm changes position and respiratory excursions during postural loading. However, it is unclear how it reacts to lifting a load while breath-holding or breathing with simultaneous voluntary contraction of the abdominal muscles (VCAM). This study analyzed diaphragm motion in healthy individuals during various postural-respiratory situations.

**Methods:**

31 healthy participants underwent examination of the diaphragm using M-mode ultrasonography, spirometry, and abdominal wall tension (AWT) measurements. All recordings were performed simultaneously during three consecutive scenarios, i.e., 1. Lifting a load without breathing; 2. Lifting a load and breathing naturally; 3. Lifting a load and breathing with simultaneous VCAM.

**Results:**

Using paired-samples *t*-tests, lifting a load without breathing displaced the diaphragm’s expiratory position more caudally (*P* < .001), with no change noted in the inspiratory position (*P* = .373). During lifting a load breathing naturally, caudal displacement of the diaphragm’s inspiratory position was presented (*P* < .001), with no change noted in the expiratory position (*P* = 0.20) compared to tidal breathing. Total diaphragm excursion was greater when loaded (*P* = .002). Lifting a load and breathing with VCAM demonstrated no significant changes in diaphragm position for inspiration, expiration, or total excursion compared to natural loaded breathing. For all scenarios, AWT measures were greater when lifting a load (*P* < .001).

**Conclusion:**

In healthy individuals, caudal displacement and greater excursions of the diaphragm occurred when lifting a load. The postural function of the diaphragm is independent of its respiratory activity and is not reduced by the increase in AWT.

## Introduction

The diaphragm is one of the most important skeletal muscles of the human body, performing vital respiratory function. The diaphragm is not only the chief inspiratory muscle but is also involved in non-respiratory activities, acting as the lower esophageal sphincter [1] and playing a crucial role in postural stabilization [2, 3]. During contraction, the central tendon of the diaphragm moves caudally and reduces intra-thoracic pressure while concurrently increasing intra-abdominal pressure (IAP) [4]. As lung volume increases above tidal volume, it correlates positively with IAP and causes stiffening of the spine [5]. Conversely, IAP decreases during tidal expiration. However, in situations requiring higher respiratory demands, IAP may increase during expiration due to abdominal muscle activity assisting in the respiratory pump [6, 7].

Adequate IAP is an essential mechanism for spinal stabilization [8]. Greatest IAP is generated in non-respiratory maneuvers when strong co-activation between the diaphragm and abdominal musculature occurs [9]. During lifting tasks, IAP correlates with the postural demands [10, 11]. Of all abdominal muscles, the transversus abdominis (TrA) activity is the most closely related to changes in the IAP [12, 13]. Several studies have demonstrated that activity of the diaphragm and TrA precedes phasic movement of the limbs as a part of the anticipatory postural adjustments [2, 14–16]. Therefore, the suboptimal reaction of the diaphragm [17, 18] and/or abdominal muscles [19, 20] to spinal loading may result in low back pain (LBP). Still, controversies exist regarding spinal stabilization mechanisms and its role in LBP. Hodges et al. [21] demonstrated through electromyography that transversus abdominis activity is delayed in individuals with LBP and suggests that the mechanism of preparatory spinal control is altered in people with LBP for movement at a variety of speeds [20]. This statement, however, is inconsistent with the results of Mehta et al. [22] who did not confirm a significant difference in trunk muscle activation and deactivation relative to arm motion between LBP subjects and healthy controls. Albeit there is no clear consensus if trunk muscle timing is an underlying mechanism of LBP as Lederman [23] states in his paper entitled “The Myth of Core Stability” that weak trunk muscles, weak abdominals, and imbalances between trunk muscles groups are not pathological, just a normal variation not leading to back pain. Also, wide variability in the degree of diaphragm contractility during breathing and postural tasks exists [24]. Therefore, we designed this study to better understand the diaphragm and abdominal muscles involvement during lifting tasks which may help to clarify controversial opinions on spinal stabilization. Based on our results, future follow-up studies may identify specific methods to improve postural stabilization in strength training and LBP treatment.

Dynamic imaging evaluating the diaphragmatic motion can be performed with different techniques, including fluoroscopy [25], dynamic MRI [17, 26, 27], EMG [28, 29], and M-mode ultrasonography [30]. There are several advantages of M-mode ultrasonography; it is a radiation-free, widely available, cost-effective, and real-time type of assessment [31]. Moreover, diaphragm excursion measurements using M-mode techniques are accurate and reproducible [32, 33].

This study investigates diaphragmatic excursions during postural tasks using M-mode ultrasonography. Unlike MRI, ultrasonography allows the assessment of diaphragmatic movement during various natural movements in a vertical posture or during more challenging activities such as lifting. On the other hand, the M-mode ultrasonography can visualize only the posterior and lateral parts of the diaphragm but cannot differentiate the costal and crural sections. Furthermore, ultrasound imaging of the diaphragm is highly operator-dependent and position-dependent, and therefore the experience of the operator is crucial for obtaining suitable images. For a more complete understanding, we combined ultrasonographic assessment with spirometry and evaluation of abdominal wall tension (AWT) by a device called DNS Brace, which is designed to indirectly monitor IAP changes [34, 35].

This prospective cross-sectional study aims to determine the changes in diaphragmatic position and to measure diaphragmatic excursions during three scenarios: (1) Lifting a load without breathing; (2) Lifting a load and breathing naturally; (3) Lifting a load and breathing with simultaneous voluntary contraction of the abdominal muscles (VCAM). The previous MRI studies analyzed postural-respiratory motion of the diaphragm in a supine position with subjects breathing naturally [17, 18, 27]. This is the first study exploring the difference in diaphragmatic excursions in a standing position while lifting a load and breathing versus breath holding, and determining how is diaphragmatic motion modified when IAP is intentionally increased with VCAM. We hypothesized that postural activity, i.e., load-lifting, would cause caudal displacement of the diaphragm during both inspiration and expiration monitored by the ultrasonography and also increased AWT monitored by the DNS brace. Additionally, we expected VCAM would reduce diaphragmatic excursions throughout an entire breathing cycle.

## Methods

### Participants

A sample of 31 subjects (average age = 28.7 ± 5.8 years) were recruited via social media to participate in the study. Demographic characteristics are summarized in Table 1. First, the examination procedures were explained, and written informed consent was signed by each subject. The exclusion criteria were: (1) low back pain, (2) previous abdominal or spine surgery, (3) respiratory or musculoskeletal disorder, (4) any symptoms of any kind of disease, (5) medical/surgical procedure or trauma within four weeks before initiation of the study, (6) pregnancy, (7) and waist to height ratio (WHtR) greater than 0.59. The study was conducted in accordance with the Declaration of Helsinki, approved by the Ethics Committee of the University Hospital Motol in Prague, Czech Republic (Approval ID: EK-237/21), and was prospectively registered at clinicaltrials.gov with trial registration number NCT04841109.

**Table 1:** Descriptive statistics of participants (mean ± standard deviation).

Participants	Age (yr)	Height (cm)	Weight (kg)	Waist Circumference (cm)	Waist/Height Ratio	Body Mass Index (kg/m^2^)
All (n = 31)	28.7 ± 5.8	173.2 ± 8.5	66.2 ± 9.2	74.1 ± 5.9	0.43 ± 0.03	22.0 ± 1.6
Males (n = 11)	28.4 ± 5.0	181.0 ± 6.0	74.1 ± 8.0	78.9 ± 6.2	0.44 ± 0.03	22.6 ± 1.7
Females (n = 20)	28.9 ± 6.4	169.0. ± 6.4	61.9 ± 6.8	71.5 ± 3.9	0.42 ± 0.02	21.6 ± 1.4

### Instrumentation

#### M-mode ultrasonography

We assessed real-time diaphragm displacement using M-mode ultrasonography with Toshiba (Canon Medical Systems Corporation, Otawara, Japan) Aplio i600 ultrasound system. The 3.5 MHz convex transducer Toshiba PVI-475BT (i8C1) was held in the right subcostal area between the mid-clavicular and anterior axillary lines, where the liver served as an acoustic window. The ultrasound beam was directed medially, cranially, and dorsally to visualize the perpendicularly posterior third of the right hemidiaphragm [36–38]. To maximize the accuracy in the probe positioning and directing, all ultrasonographic examinations were performed by the same examiner, who has achieved 32 years’ experience. In the M-mode image, the diaphragm appears as a single echogenic line that moves towards the probe during inspiration and away from the probe during expiration. The diaphragmatic position was evaluated by measuring its vertical distance from the baseline in mm [39]. A smaller number in mm represents a more caudal position of the diaphragm. The excursions were then calculated as a difference between the end-inspiratory and the end-expiratory position within one breathing cycle.

#### DNS brace

To correlate the postural function of the diaphragm with abdominal muscle tension, we simultaneously measured AWT using a device called DNS Brace, described in previous studies [34, 35]. A recent study [35] demonstrated strong correlations exist between AWT measured by the DNS Brace and IAP measured via anorectal pressure using high-resolution manometry; meaning the DNS Brace is a valid tool to monitor changes in IAP accurately. Before the examination, all four sensors attached to DNS Brace were calibrated to 0 kilopascals (kPa) in the tidal end-expiratory phase of the breathing cycle.

#### Spirometry

This investigation also included spirometry, which for the purposes of this study was primarily used to define the end-inspiratory, and end-expiratory phases of the breathing cycle, as well as to confirm the breath-holding state. Spirometric measurements were performed using a portable spirometer Jaeger MasterScope (VIASYS Healthcare, Hoechberg, Germany).

### Procedures

All study procedures were conducted in the same examination room and under the same conditions. Participants were requested not to eat for at least 1.5 hours before the procedure. The examination consisted of three consecutive scenarios wherein ultrasonographic, spirometric, and AWT measurements were performed simultaneously. The assessment was performed by the same examiners in order to reduce inter-observer variability and repeated three times to reduce intra-observer variability. All data were stored for subsequent analysis, and the average value for each measurement was calculated.

**Fig 1:**
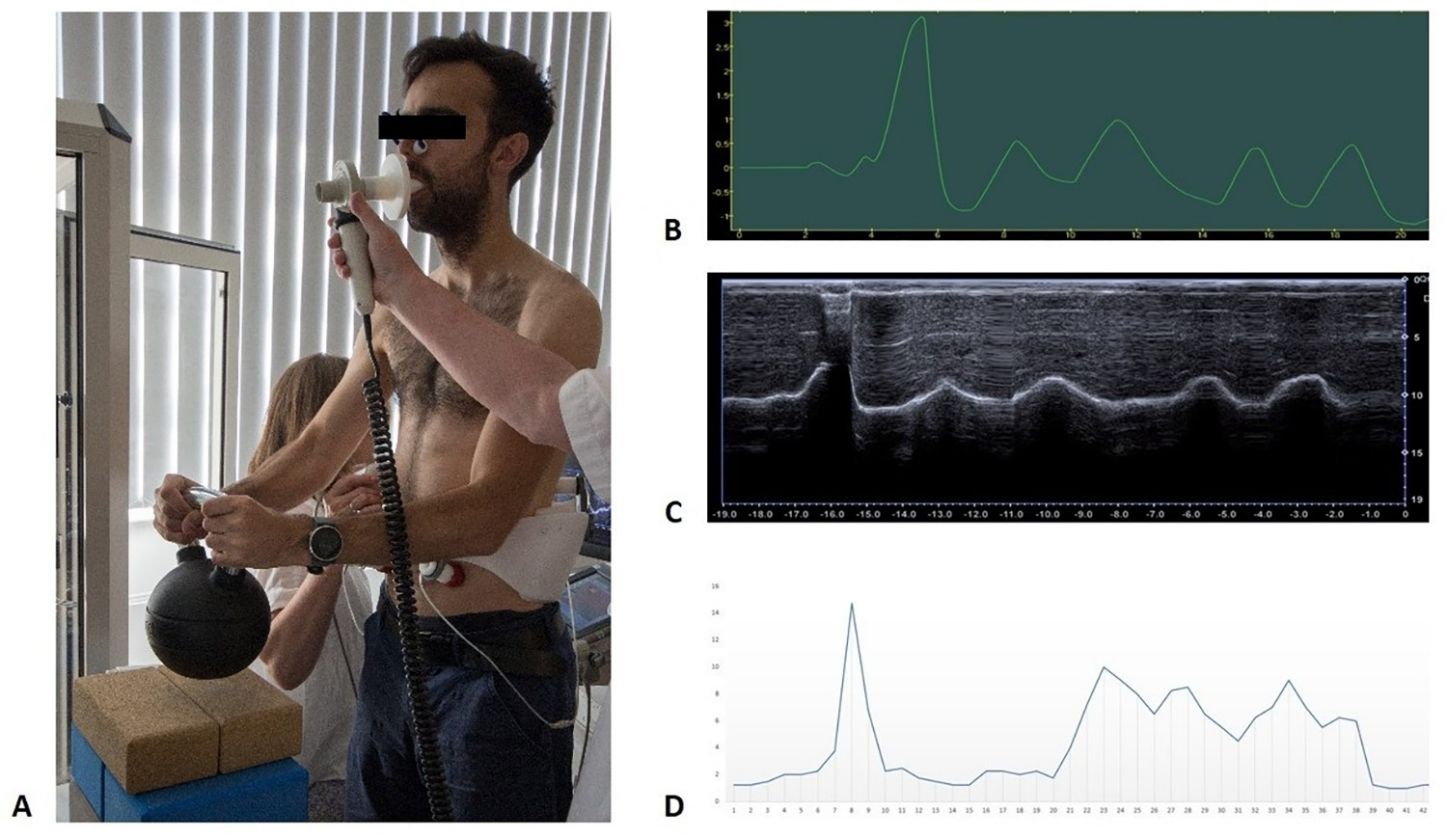
Examination procedures. (**A**) Participant assessed simultaneously by M-mode ultrasonography, spirometry, and DNS brace. (**B**) Spirometry record displayed in Grapher software (**C**) M-mode view of diaphragm motion. (**D**) Graphical representation of recorded data from DNS Brace. All of these recordings were taken during the same examination of one participant in Scenario 2.

Subjects were assessed in a standing position with their feet shoulder-width apart. Their arms were close to the body with the elbows bent, and their hands were placed on the kettlebell handle. The kettlebell weight was approximately 20% (18–22%) of the participant’s body weight with the lightest kettlebell weighing 10 kg and the heaviest weighing 18 kg. The participant was instructed to lift and hold the kettlebell only by bending the elbows without tilting the body. The DNS brace was tightly fitted to the participant’s body with two front sensors bilaterally placed above the groin and two back sensors to the trigonum lumbale in the dorsolateral part of the abdominal wall. Subjects breathed through a mouthpiece connected to a pneumotachograph and used a nasal clip to preclude air leakage. The ultrasonographic probe was held under their right arm in the subcostal region and maneuvered to obtain the clearest image of the posterior third of the diaphragm (see Fig 1). Prior to the examination, each subject inhaled and exhaled several times to check that all instruments were measuring correctly. All examiners were blinded to the results of other assessments.

Each scenario was initiated by deep inspiration followed by a rapid forced expiration that served as a time marker to synchronize all the recordings. One recording lasted no longer than 20 seconds. The scenarios were as follows:

Scenario 1 started with one tidal inspiration and expiration. The participant then inhaled and held the breath at the end of the inspiratory phase followed by lifting and lowering the kettlebell, after that exhaled and held the breath at the end of the expiratory phase while lifting and lowering the kettlebell.

Scenario 2 consisted of two tidal inspirations and expirations, then the kettlebell was lifted, followed by two loaded inspirations and expirations while the kettlebell was held.

Scenario 3 consisted of two tidal inspirations and expirations, then the participant intentionally contracted the abdominal muscles and lifted the kettlebell, followed by two loaded inspirations and expirations while holding the kettlebell and having the abdominal wall tensed.

### Statistical analysis

Descriptive statistics were calculated for all variables. Data are mean ± standard deviation (SD), unless otherwise stated. Reliability of the ultrasonography and DNS Brace measures were calculated from the average measurements of two tidal breaths recorded at different time points for each subject. Intraclass correlation coefficient estimates (ICC2, k), 95% confidence intervals, and standard error of measurement (SEM) were calculated from tidal inspiration and expiration, separate from the different scenarios, and are presented in Table 2. The ICC’s were calculated based on a mean-rating (k = 3), absolute-agreement, 2-way mixed-effects model. Reliability was interpreted as poor (< 0.5), moderate (0.5–0.75), good (0.75–0.9), and excellent (> 0.9) [40]. Paired-samples *t*-tests were used to determine changes in the diaphragm position and AWT for each scenario described, with effect sizes interpreted as *small* (< 0.2), *medium* (0.5), or *large* (> 0.8) as proposed by Cohen [41]. Power analysis, using G*Power 3.1, indicated an 80% chance of detecting a medium effect size of 0.5 in 27 subjects with statistical significance determined a priori at *p* < 0.05 (one-tailed). With highly correlated variables, Bonferroni corrections were utilized when testing multiple hypotheses. All data analyses were conducted using the Statistical Package for the Social Sciences (SPSS version 28.0 for Mac; IMB Corp, Armonk, NY).

**Table 2:** Intraclass correlation coefficients of ultrasonography and DNS brace values during tidal inspiration and expiration (ICC _2, k_).

			95% Confidence Interval			F Test With True Value 0		
Measure		ICC	Lower Bound	Upper Bound	SEM	Value	*df*1	Sig
Ultrasonography	Inspiration	.985**	.970	.993	1.68	71.63	30	< .001
	Expiration	.989**	.978	.995	1.54	93.40	30	< .001
DNS Brace	Inspiration	.657*	.291	.834	0.82	2.90	30	.002
	Expiration	.569*	.136	.789	0.78	2.57	30	.006

Note: ICC = Intraclass Correlation Coefficient

SEM = Standard Error of Measurement

**Denotes: Excellent reliability

*Denotes: Moderate reliability

## Results

### Preliminary analyses

Univariate outliers were assessed by calculating z-scores for each of the dependent variables using complete data for all scenarios (n = 31). Normality was assessed using ± 1.96 as the cutoff for the absolute z-score skew and kurtosis (respectively) for each variable [42]. Results evidenced one variable that was not normally distributed, and three scores considered to be outliers with absolute z-score values greater than the recommended cutoff value of 3.29 (one in Scenario 2, and two in Scenario 3). These outliers did not occur on the same variables, and were handled by winsorization; where the outlier retained its rank value and was replaced with the next largest value [43]. This process improved overall normality to within acceptable ranges, with no absolute z-scores larger than ± 1.96 for skew or kurtosis after correction of the outliers.

### Hypothesis testing

**Fig 2:**
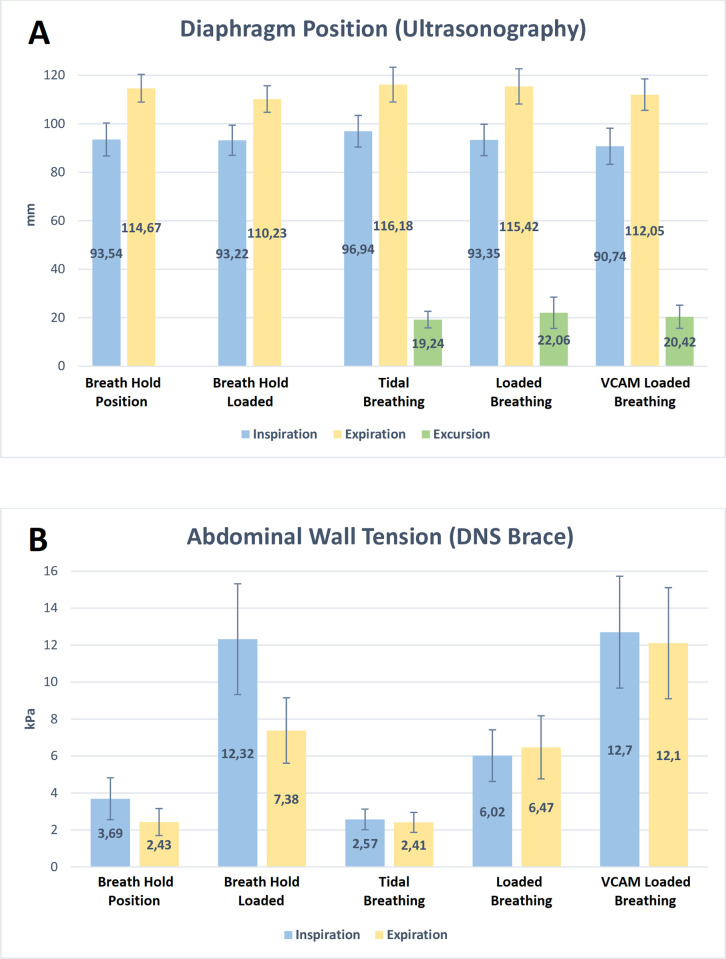
Graph showing outcomes of each scenario. (**A**) Diaphragm position, excursions (mm), and (**B**) abdominal wall tension (kPa) during breath-holding, tidal breathing, loaded breathing, and loaded breathing with VCAM (Mean ± Standard Deviation).

Table 3 displays the means ± standard deviation (SD), mean differences, and outcomes of each scenario. These data are presented graphically in Fig 2.

**Table 3:** Changes in ultrasonography values (mm) and DNS brace values (kPa) during different scenarios of holding a load equivalent to 20% body weight (mean [standard deviation]).

**Scenario 1** ^**a**^	**Measure**	**Breath Hold Beginning Position**	**Breath Hold Loaded Position**	**Mean Difference**	**95% CI**	**Effect Size **	***P* V*alue***
Ultrasonography	Inspiration	93.54 (13.59)	93.22 (12.41)	0.32	(-1.69, 2.33)	0.06	0.373
	Expiration	114.67 (11.40)	110.23 (10.95)	4.45	(2.96, 5.93)	1.1	< .001*
DNS Brace	Inspiration	3.69 (2.27)	12.32 (5.99)	-8.63	(-10.65, -6.61)	-1.57	< .001*
	Expiration	2.43 (1.46)	7.38 (3.54)	-4.95	(-6.17, -3.73)	-1.49	< .001*
**Scenario 2** ^**b**^	**Measure**	**Tidal Breathing**	**Loaded Breathing**	**Mean Difference**	**95% CI**	**Effect Size **	***P* V*alue***
Ultrasonography	Inspiration	96.94 (13.03)	93.35 (12.92)	3.58	(2.07, 5.09)	0.87	< .001**
	Expiration	116.18 (14.35)	115.42 (14.51)	0.76	(-0.42, 1.95)	0.24	0.201
DNS Brace	Inspiration	2.57 (1.12)	6.02 (2.80)	-3.44	(-4.45, -2.44)	-1.26	< .001**
	Expiration	2.41 (1.08)	6.47 (3.41)	-4.06	(-5.23, -2.89)	-1.28	< .001**
Ultrasonography	Excursion	19.24 (6.85)	22.06 (8.79)	-2.82	(-4.48, -1.16)	-0.63	.002**
**Scenario 3** ^**c**^	**Measure**	**Loaded Breathing**	**VCAM Loaded Breathing**	**Mean Difference**	**95% CI**	**Effect Size **	***P* V*alue***
Ultrasonography	Inspiration	93.35 (12.92)	90.74 (14.89)	2.61	(-0.26, 5.49)	0.33	0.037
	Expiration	115.42 (14.51)	112.05 (12.93)	3.36	(0.05, 6.68)	0.37	0.023
DNS Brace	Inspiration	6.02 (2.80)	12.70 (6.05)	-6.68	(-8.26, -5.09)	-1.55	< .001**
	Expiration	6.47 (3.41)	12.10 (6.01)	-5.63	(-7.49, -3.77)	-1.11	< .001**
Ultrasonography	Excursion	22.06 (8.79)	20.42 (9.54)	1.65	(-0.97, 4.26)	0.23	0.104

^a^Subject holds breath at the end of full Inspiration and Expiration

^b^Subject holds load with natural breathing and spontaneous abdominal activity

^c^Subject voluntarily contracts abdominal muscles prior to holding load while breathing naturally

Note: VCAM: Voluntary contraction of abdominal muscles

Effect size = calculated Cohen’s d

*Statistically significant difference observed (Bonferroni Correction *P* < 0.025)

**Statistically significant difference observed (Bonferroni Correction *P* < 0.016)

#### Scenario 1

For Scenario 1, ultrasonography demonstrated the inspiratory breath-hold position of the diaphragm (mm) was not statistically different from inspiration when holding the weight (*t*(30) = .327, *p* = 0.37), but the DNS Brace demonstrated significantly greater AWT values (kPa) during inspiration when holding the weight (*t*(30) = -8.27, *p* < .001). During expiration, ultrasonography demonstrated the expiratory breath-hold position of the diaphragm was significantly different when holding the weight (*t*(30) = 6.13, *p* < .001), and the DNS Brace also demonstrated significantly greater AWT values (kPa) during expiration when holding the weight (*t*(30) = -8.27, *p* < .001).

#### Scenario 2

For Scenario 2, ultrasonography demonstrated the loaded inspiratory breath position of the diaphragm (mm) was significantly lower, i.e. more caudal, when holding the weight compared to tidal breathing (*t*(30) = 4.84, *p* < .001.), but the loaded expiratory position was not significantly different from tidal breathing (*t*(30) = 1.31, *p* = 0.10). The total excursion of the diaphragm (difference between expiratory and inspiratory values in mm) was significantly greater during loaded breathing compared to tidal breathing (*t*(30) = 3.48, *p* = .002). The DNS Brace demonstrated significantly greater AWT values (kPa) during both loaded inspiration (*t*(30) = -7.0, *p* < .001.) and loaded expiration (*t*(30) = -7.10, *p* < .001.) compared to tidal breathing.

#### Scenario 3

For Scenario 3, ultrasonography demonstrated the VCAM loaded position of the diaphragm (mm) was not significantly different from the spontaneous loaded breathing position for inspiration (*t*(30) = 1.86, *p* = .037) or expiration (*t*(30) = 2.07, *p* = .023) after a Bonferonni correction. The total VCAM excursion of the diaphragm (difference between expiratory and inspiratory values in mm) was also not significantly different from the spontaneous loaded excursion (*t*(29) = 1.29, *p* = .104). The DNS Brace demonstrated significantly greater AWT values (kPa) during both VCAM loaded inspiration (*t*(30) = -8.62, *p* < .001.) and expiration (*t*(30) = -6.19, *p* < .001.), compared to spontaneous loaded breathing.

## Discussion

This study demonstrated that when lifting a load, the diaphragm descends caudally and increases its excursions while abdominal wall tension significantly increases as well. Voluntary contraction of the abdominal wall does not reduce diaphragmatic excursions and does not influence its position during respiration compared to natural loaded breathing. Our results add to the current knowledge on spinal stabilization mechanisms.

During tidal breathing, the increase in IAP is not sufficient to improve spinal stiffness [5] as the force exerted by the diaphragm reaches only about 10% of its maximum [44]. IAP rises with inspiratory volume and is highest during maximal inspiratory effort [5, 45]. Since the abdominal contents are essentially incompressible, all diaphragmatic displacements must be met by equal displacements of the anterolateral abdominal wall and vice versa [46]. With increased ventilation, the abdominal muscles are engaged in expiration, rising IAP [4, 47]. Because abdominal muscles have an antagonistic respiratory function to the diaphragm, their contraction displaces the diaphragm cranially to deflate the lungs [48]. Dual postural-respiratory activity has been confirmed in both the diaphragm and TrA [2, 3]. The position of the diaphragm is determined by the balance between its contraction and the tension of the abdominal wall muscles [49–51]. When postural demands increase, the abdominal muscles and diaphragm must contract synergistically to achieve adequate IAP for spinal stabilization. It must also be emphasized that not only the diaphragm and abdominal muscles are involved in spinal stabilization, but also pelvic floor muscles [52], paraspinal muscles, and deep intrinsic back muscles such as rotatores and multifidus [53, 54]. The involvement of these muscles was not directly explored in this study.

To our knowledge, a stand-alone postural displacement of the diaphragm has not been reported in previous studies. Hodges and Gandevia [2] showed that the movement of the upper limb during breath-holding at the end of expiration activated the diaphragm, assessed by EMG. Displacement of the diaphragm without breathing was demonstrated by Kolar [26] during a maneuver in which the abdominal wall was pressurized and expanded. Using MRI and EMG diaphragmatic recordings Kolar’s team [26] demonstrated that individuals are capable of moving their diaphragm voluntarily, and that non-respiration diaphragm activity is subject of voluntary control but the amplitude of movement differs from person to person. In Scenario 1, we found that during lifting a load without breathing, caudal displacement of the diaphragm significantly occurred in the expiratory position. This finding shows that the postural displacement of the diaphragm is fully independent of its respiratory activity. In the inspiratory position, no significant displacement of the diaphragm was noted, which suggests that at the end of tidal inspiration, the diaphragm was already contracted enough to ensure stabilization of the spine in relation to the given load. This may indicate that postural contraction of the diaphragm can be substituted to some extent by inspiratory activity to achieve the required increase in IAP. Our observation is in line with studies reporting increased inspiratory volume depending on the magnitude of the load lifted, with inspiration occurring immediately prior to lifting as a consistent pattern of natural breath control during lifting tasks [11, 55–57].

In Scenario 2, the diaphragm moved significantly more caudally in the inspiratory phase of loaded breathing compared to tidal breathing. For loaded expiration, there was no significant change, and the diaphragm ascended similarly to tidal breathing. However, AWT was significantly greater during loaded expiration than during loaded inspiration. This indicates that the diaphragm is more involved in the IAP increase during inspiration, whereas the abdominal muscles are more active during expiration in accordance with their respiratory function. This is in line with the findings of Hodges and Gandevia [3], where the amplitude of diaphragm EMG was higher in inspiration than in expiration, while TrA activity was the opposite. This further supports results noted by Kolar et al. [27], where dynamic MRI was used to measure diaphragmatic displacement in the supine position while inducing postural activity by the pressure of the upper and lower extremities against resistance. They showed significantly more caudal diaphragmatic displacement during inspiration with isometric postural limb activities than during expiration with isometric limb activity. Additionally, they also found no significant change in the expiratory position of the diaphragm when using the upper limbs for postural loading. Thus, it appears that postural functions further challenge the diaphragm during inspiration, and the abdominal muscles are further challenged during expiration, which should be considered when targeting specific muscles during postural training.

Hodges et al. [29] investigated the postural-respiratory function of the diaphragm and other muscles of the trunk with higher respiratory demands. These researchers reported that respiratory function was superior to postural function because EMG activity of the diaphragm and TrA declined after one minute of increased ventilation, along with a reduction in IAP. In our study, the priority of the respiratory function of the diaphragm during postural loading is evident in Scenario 3. We expected that the intentional increase in IAP evoked by VCAM would reduce the inspiratory displacement of the diaphragm. Surprisingly, the VCAM loaded breathing was not significantly different from natural loaded breathing, although there was about a twofold increase in VCAM AWT. We speculate that the diaphragm increases its contractility to overcome the higher IAP when the abdominal muscles are more tense to maintain sufficient ventilation. Therefore, it can be assumed that greater contraction of the abdominal muscles within the stabilizing function induces stronger diaphragmatic activity. Our findings support the hypothesis that weight-lifting, which results in greater IAP, provides a strength-training stimulus for the diaphragm [58, 59]. This tightly coupled muscle function has also been observed in reverse, a study examining inspiratory resistance-training of the diaphragm noted increased thickness of not only the diaphragm but also the TrA [60]. Hence, VCAM could be beneficial as part of postural-respiratory training, especially for people with LBP presenting with higher diaphragm positions [17, 18] and smaller diaphragm excursions [17, 18, 61], which is compensated by greater inspiration within the lungs to secure adequate IAP when performing lifting tasks [62]; or for athletes with lumbopelvic pain who have been shown to have a reduced diaphragm thickness [63]. This is further supported by Finta et al. [60], who confirmed the combination of trunk and diaphragm strengthening as a viable therapeutic approach for chronic nonspecific LBP.

Further research is needed to investigate the movement of the diaphragm and AWT under higher loads both in healthy individuals and subjects with LBP or respiratory diseases.

### Limitations

There were some limitations in our study. First, the lifted load corresponded to approximately 20% of the subject’s body weight. For higher loads, most subjects were unable to lift the weight with only elbow flexion while not tilting the body, which was essential for stable ultrasonography imaging.

We attempted to eliminate potential measurement error caused by uneven probe pressure by repeating each assessment three times for each subject. Another possible error could arise from an increase in AWT, which could conceivably lengthen the distance of the probe from the diaphragm. This was not shown to be the case in either scenario. In all scenarios during lifting a load, the distance of the diaphragm from the probe remained equal or approached compared to the unloaded situations. There may be minor inaccuracies in ultrasound imaging, but the m-mode ultrasonography of the diaphragm is generally considered to be a reliable and accurate type of assessment [32, 33].

The DNS brace, although adjustable, may not fit on everyone’s body in the same way and therefore measure AWT activity with all sensors identically. This measurement variability was reduced by calibrating before the actual measurement and calculating the average pressure from all four sensors.

When interpreting the results, also the role of fatigue or motor learning resulting from the 3 repetitions of each test should be considered. However, since each sequence was of only 20 seconds duration, with a pause after each trial and no subject complained of fatigue nor commented on any execution change when repeating the test, we do not think these factors were of relevant importance.

We did not analyze the data by gender due to the limited number of participants and numerical disproportion between men and women. This could be a goal of future studies since sex differences in diaphragmatic excursions [33, 36, 64] were reported in some studies. We have also measured spirometry data which are not included in this manuscript as they would be beyond the scope of this paper. Spirometry data will be presented in a subsequent article.

## Conclusion

This study investigated how the position of the diaphragm changes during breathing and breath-holding while lifting a load. The second aim was to clarify how the voluntary contraction of the abdominal wall resulting in IAP increase modifies diaphragmatic position and excursions.

Our findings demonstrate that a postural task, such as load-lifting, induces caudal displacement of the diaphragm independently of respiration in breath-holding situations. This study also confirms that the diaphragm moves significantly more caudally during the inspiratory phase of breathing and increases its excursions when loaded. However, it was not proven that VCAM reduces diaphragm motion, despite a twofold increase in AWT compared to natural loaded breathing.

## Supporting information

S1 Dataset De-identified dataset for Scenario 1. (XLSX)

S2 Dataset De-identified dataset for Scenario 2. (XLSX)

S3 Dataset De-identified dataset for Scenario 3. (XLSX)
